# Supplementary material for: Novel CRISPR/Cas9 gene drive constructs reveal insights into mechanisms of resistance allele formation and drive efficiency in genetically diverse populations
Source: PLoS Genet. 2017 Jul 20;13(7):e1006796. doi: 10.1371/journal.pgen.1006796 (PMC5518997; doi:10.1371/journal.pgen.1006796)
Supplement: S1 Fig — (A) In a homozygous female with genotype D/D, high expression of Cas9 results in relatively high formation of resistance alleles (~30%) in early female embryos after fertilization by a wild type male due to persistence of maternally expressed Cas9. (B) In a heterozygous female with genotype D/r2, no drive conversion takes place in the germline, and lower expression of Cas9 results in reduced formation of resistance alleles (~20%) in female embryos after fertilization by a wild type male. (C) In a male with the gene drive, no drive conversion takes place in the germline due to the presence of only one X-chromosome. Additionally, the relatively small size of the gamete means that a significant amount of Cas9 does not persist to the embryo, resulting in little to no post-fertilization formation of resistance alleles. Note that any formation of resistance alleles in the embryo may result in mosaicism of adult individuals, as we frequently observed in our crosses. Additionally, leaky expression of Cas9, as observed in the vasa construct, may potentially form additional resistance alleles in the embryo or later stages. Tables 1–3 in S1 Dataset provide the calculations used for inference of these rates from the phenotypes of progeny in our crosses. (PDF) [file pgen.1006796.s001.pdf]

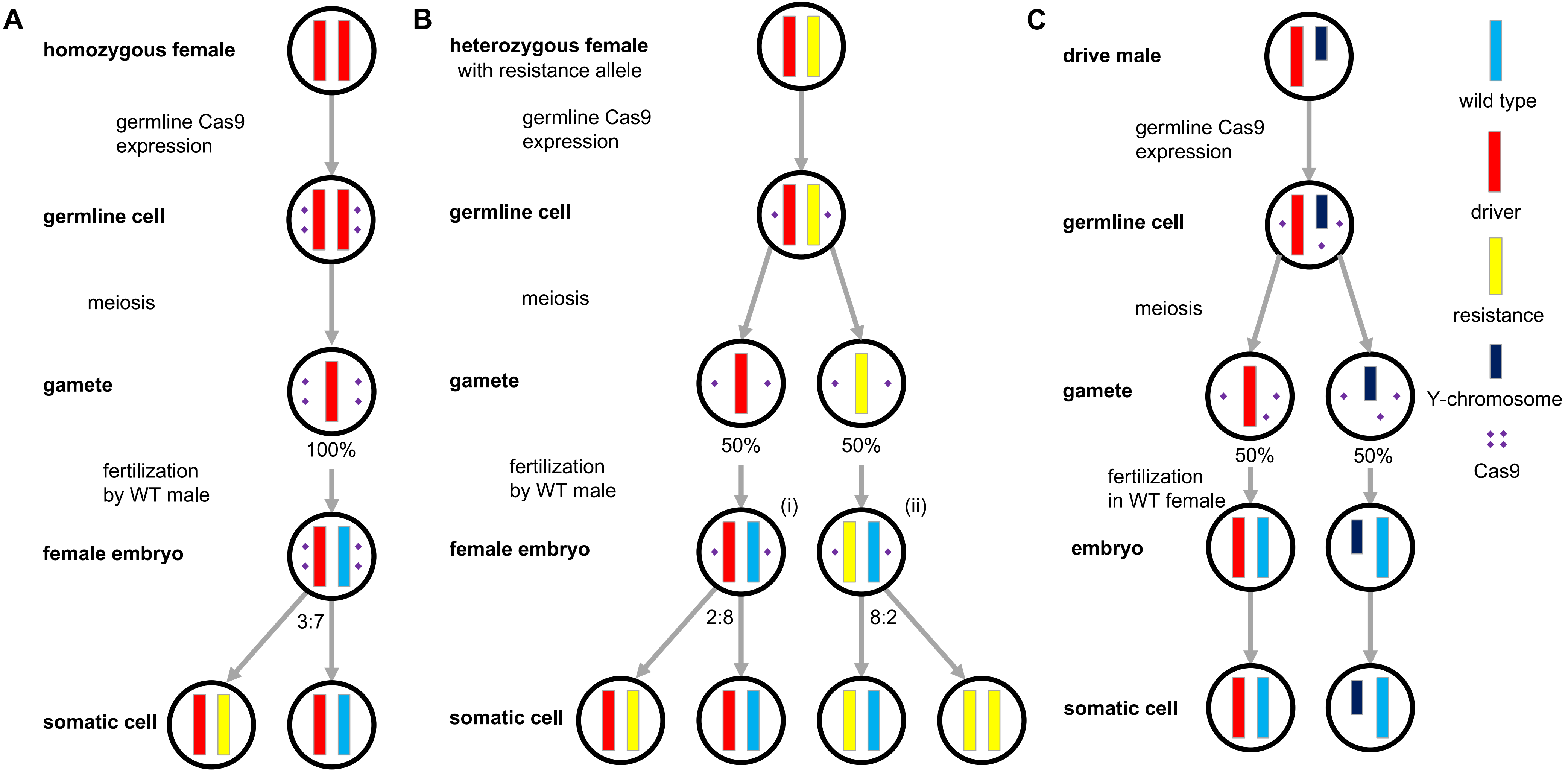

A
homozygous female
germline Cas9
expression
germline cell
meiosis
gamete
100%
fertilization
by WT male
female embryo
3:7
somatic cell
B
heterozygous female
with resistance allele
germline Cas9
expression
germline cell
meiosis
gamete
50%
50%
fertilization
by WT male
(i)
(ii)
female embryo
2:8
8:2
somatic cell
C
drive male
germline Cas9
expression
germline cell
meiosis
gamete
50%
50%
fertilization
in WT female
embryo
somatic cell
wild type
driver
resistance
Y-chromosome
Cas9
