# Supplementary figures and images for: Novel CRISPR/Cas9 gene drive constructs reveal insights into mechanisms of resistance allele formation and drive efficiency in genetically diverse populations
Source: PLoS Genet. 2017 Jul 20;13(7):e1006796. doi: 10.1371/journal.pgen.1006796 (PMC5518997; doi:10.1371/journal.pgen.1006796)

# Germline Event Type Correlation

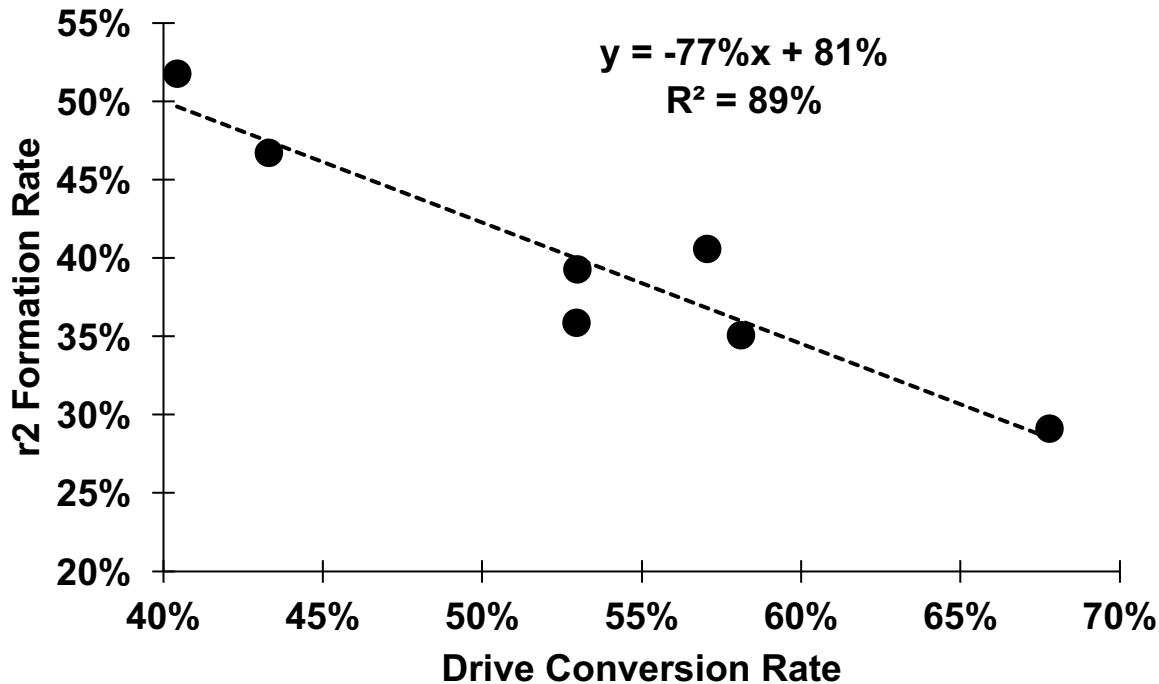

Supplement: S2 Fig — All rates were obtained from Table 3 in S1 Dataset. (PDF) [file pgen.1006796.s002.pdf]
