# Supplementary material for: Novel CRISPR/Cas9 gene drive constructs reveal insights into mechanisms of resistance allele formation and drive efficiency in genetically diverse populations
Source: PLoS Genet. 2017 Jul 20;13(7):e1006796. doi: 10.1371/journal.pgen.1006796 (PMC5518997; doi:10.1371/journal.pgen.1006796)
Supplement: S1 Appendix — (PDF) [file pgen.1006796.s003.pdf]

S1 Appendix

Plasmid construction:

The following tables show the DNA fragments used for Gibson Assembly of the listed plasmids. PCR products are shown with the oligonucleotide primer pair used, and plasmid digests are shown with the restriction enzymes used.

**IHDyi1**	*Template*	*Oligo/Enzyme 1*	*Oligo/Enzyme 2*
PCR Product	Genomic DNA	YellowLeft_F	YellowLeft_R
Plasmid Digest	pDsRed-attp	*Eco*RI-HF	*Nru*I-HF

**IHDypi1**	*Template*	*Oligo/Enzyme 1*	*Oligo/Enzyme 2*
PCR Product	Genomic DNA	YellowPLeft_F	YellowPLeft_R
Plasmid Digest	pDsRed-attp	*Eco*RI-HF	*Nru*I-HF

**IHDyg1**	*Template*	*Oligo/Enzyme 1*	*Oligo/Enzyme 2*
PCR Product	none	Yellow_gRNA_F	Yellow_gRNA_R
Plasmid Digest	pCFD3	*Bbs*I	none

**IHDypg1**	*Template*	*Oligo/Enzyme 1*	*Oligo/Enzyme 2*
PCR Product	none	YellowP_gRNA_F	YellowP_gRNA_R
Plasmid Digest	pCFD3	*Bbs*I	none

**IHDyi2**	*Template*	*Oligo/Enzyme 1*	*Oligo/Enzyme 2*
PCR Product	IHDyg1	YellowU6_3_gRNA1_F	YellowU6_3_gRNA1_R
PCR Product	Genomic DNA	YellowRight_F	YellowRight_R
Plasmid Digest	IHDyi1	*Pst*I-HF	*Spe*I-HF

**IHDypi2**	*Template*	*Oligo/Enzyme 1*	*Oligo/Enzyme 2*
PCR Product	IHDypg1	YellowU6_3_gRNA_F	YellowPU6_3_gRNA_R
PCR Product	Genomic DNA	YellowPRight_F	YellowPRight_R
Plasmid Digest	IHDypi1	*Spe*I-HF	*Xho*I

**IHDyN1**	*Template*	*Oligo/Enzyme 1*	*Oligo/Enzyme 2*
PCR Product	pnos-Cas9-nos	NosCas9_1_F	NosCas9_1_R
PCR Product	pnos-Cas9-nos	NosCas9_2_F	NosCas9_2_R
Plasmid Digest	IHDyi2	*Not*I-HF	*Sph*I-HF

**IHDypV1**	*Template*	*Oligo/Enzyme 1*	*Oligo/Enzyme 2*
PCR Product	pvasa-Cas9	VasaCas9_1_F	VasaCas9_1_R
PCR Product	pvasa-Cas9	VasaCas9_2_F	VasaCas9_2_R
Plasmid Digest	IHDypi2	*Not*I-HF	*Sph*I-HF

IHDyV1*	*Template*	*Oligo/Enzyme 1*	*Oligo/Enzyme 2*
PCR Product	pvasa-Cas9	VasaCas9_1_F	VasaCas9_1_R
PCR Product	pvasa-Cas9	VasaCas9_2_F	VasaCas9_2_R
Plasmid Digest	IHDyi2	*Not*I-HF	*Sph*I-HF

*Not used in the study, but included for completeness

Construction primer list:

```
NosCas9_1_F:ACTACGATCGCAGGTGTGCATATGTCCGCGGCCGCGCTTCGACCGTTTTAACCTCGAAAT
NosCas9_1_R:TCCTGTATATCGGCGCCTCTT
NosCas9_2_F:AAGAGGCGCCGATATACAGGA
NosCas9_2_R:GCTGTGGGTTTTGGACACTGGGAATTCTTGCATGCTCCTTCCTGGCCCTTTTCGAG
VasaCas9_1_F:ACTACGATCGCAGGTGTGCATATGTCCGCGGCCGCCTGCAGCTGGTTGTAGGTGCAGTTG
VasaCas9_1_R:GTTCCTCGGTGCCGTCCATCTTTTC
VasaCas9_2_F:GAAAAGATGGACGGCACCGAGGAAC
VasaCas9_2_R:GGGTTTTGGACACTGGGAATTCTTGCATGGCTAGCCAACACGAAGAGCAGCAGTGTGGTG
Yellow_gRNA_F:TATATATAGACCTATTTTCAATTTAACGTCGGTTTTGGACACTGGAACCG
Yellow_gRNA_R:ATTTTAACTTGCTATTTCTAGCTCTAAAACCGGTTCCAGTGTCCAAAACC
YellowLeft_F:AATTAACCAATTCTGAACATTATCGCCTAGGGTTGCGAGGTTTTAGGACTGAAAGAGCAC
YellowLeft_R:TGCATATGTCCGCGGCCGCTAGCATGCAAGAATTCTTCCAGTGTCCAAAACCCACAGCC
YellowP_gRNA_F:TATATATAGACCTATTTTCAATTTAACGTCGATGATTCACAATTCACTGA
YellowP_gRNA_R:ATTTTAACTTGCTATTTCTAGCTCTAAAACTCAGTGAATTGTGAATCATC
YellowPLeft_F:CCAATTAACCAATTCTGAACATTATCGCCTAGGGCGCTCCGCTTGATGTTGTTTTGTT
YellowPLeft_R:TATGTCCGCGGCCGCTAGCATGCAAGAATTCTGAGGGTCAAATATTTGGTTTCCGCTAGT
YellowPRight_F:GCATCCTGCAGGACGCGTGTGAATTGTGAATCATCGGTGACGCC
YellowPRight_R:AACTCGATTGACGGAAGAGCCTCGAGCACACAGTGCACAAGGATCCACCCTTTGTCCTGG
YellowPU6_3_gRNA_R:TTCACAATTCACACGCGTCCTGCAGGATGCATACGCATTAAGCGAACATT
YellowRight_F:GCATCCTGCAGGACGCGTCCGTGGGCATCGGCAATACCACC
YellowRight_R:GATTGACGGAAGAGCCTCGAGCTGCACACACAGTGGACTACATTGCCTGAATTGGCGGGC
YellowU6_3_gRNA_F:ATGTATGCTATACGAAGTTATAGAAGAGCACTAGTTTTTTTGCTCACCTGTGATTGCTC
YellowU6_3_gRNA_R:CGATGCCCACGGACGCGTCCTGCAGGATGCATACGCATTAAGCGAACATT
```

Sequencing primer list:

```
Cas9_S1_F:CTGCTACCTGCAGGAGATCTTT
Cas9_S1_R:TAGATCAACCGCAAGTCAGCC
Cas9_S2_F:CTCAGGCGGCAAGAGGATTTC
Cas9_S2_R:CCCCCTTATCCACGACTTCCT
Cas9_S3_F:GGAAGGCATAAGCCCGAGAAT
Cas9_S3_R:TGCCGTTCTGCAGGTAGTACA
Cas9_S4_F:GACAGTCCGGAAGGTCCTGT
Cas9_S4_R:TTCTCCACTTTGGCCACAACC
dsRed_S_F:ctgaagggcgagatccacaag
dsRed_S_R:GTGGGAGGTGATGTCCAGCTT
gRNA1_S_F:TTGCTCACCTGTGATTGCTCC
IHD_S_F:GGGTTATTGTCTCATGAGCGG
IHD_S_R:TCTCGAAAATAATAAAGGGAAAATCAG
Nos3_S_F:TGGCGCGTTCGATTTTAAAGAG
Nos3_S_R:TATCGGCCACGACGATTGAAC
Nos5_S_F:TGTAATTCCTGCCGCGAAACA
```

Nos5_S_R:CCGTCACAACACAATCACTGC
SV40_S_F:GGAACCTTACTTCTGTGGTGTGA
SV40_S_R:TCTGAAGGAAAGTCCTTGGGGT
Vasa_S1_F:ACTTGGCGCACTTGAGGAGAT
Vasa_S1_R:CAAGCAGCTGGCCAAAATGAC
Vasa_S2_F:CGGAAGCCGGGCTATTTCTTT
Vasa_S2_R:TGTATTAGCCATGGGAAGGCAT
Vasa_S3_F:GGCACATACCAGACAACCAGG
Vasa_S3_R:ACGTTAAGCAACCAATTTAAGGGC
VasaCas9_S1_F:TTCAGCAACGAGATGGCCAAG
VasaCas9_S1_R:GGTGCTGTCCACCAGTTTCTT
VasaCas9_S2_F:CACCAGATCCACCTGGGAGAG
VasaCas9_S2_R:GTTCCAGGGGGTGATGGTTTC
VasaCas9_S3_F:CCCGAGAACATCGTGATCGAA
VasaCas9_S3_R:AGGTACAGCTTCTCGTTCTGC
VasaCas9_S4_F:CTGAGCATGCCCCAAGTGAAT
VasaCas9_S4_R:CCACCACCAGCACAGAATAGG
VasaCas9_S5_F:TACCCTGACCAATCTGGGAGC
VasaCas9_S5_R:CCCAGCTGAGACAGGTCGAT
YellowLeft_S_F:AGAGCCATTAGCACGGCAGTTACCA
YellowRight_S_R:TCGAATGGGCGAAAGGGACATACCA
YelProLeft_S_F:ATTACCCACTTAGGGCACCCCCAAC
YelProRight_S_R:CAGTGTTCATCTTTATCGGCGACTGCAA

Resistance allele sequences from the *nanos* drive:

Red = gRNA Target Site
Orange = PAM
Blue = Insertion
- = deletion

Wild type allele
GGGTTTTGGACACTGGAA CCGTGGGCATCGGC

Wild type male alleles
Mother Batch A
GGGTTTTGGACACTGGAA CCGTGGGCATCGGC @1 wild type
GGGTTTTGGACACTGGAA C -----------CGGC x2 in-frame
GGGTTTTGGACACTGGAA CTA ----------TCGGC in-frame
Mother Batch B
GGGTTTTGGACACTGG-- --------CATCGGC x2 in-frame
GGGTTTTGGACACTGGAA CCG CCGTGGGCATCGGC in-frame
Mother Batch C
GGGTTTTGGACACTGGAA CCGTGGGCATCGGC x2 @1 wild type
GGGTTTTGGACACTGG-- GCATTTTG CCGTGGGCATCGGC in-frame
Mother Batch D
GGGTTTTGGACACTGGAA ----------TCGGC in-frame
GGGTTTTGGACACTGGAA CTCCTGGACACTCCA CCGTGGGCATCGGC in-frame
Mother Batch E
GGGTTTTGGACACTGGAA TCGTGGGATTGGACA CCGTGGGCATCGGC in-frame

Yellow phenotype male alleles
Mother Batch A (1)

```
GGGTTTTGGACA------                          CCGTGGGCATCGGC x2 in-frame
GGGTTTTGGACACTGGA-                          CCGTGGGCATCGGC x4 @1
GGGTTTTGGACACTGGA- CA                       CCGTGGGCATCGGC x3 @2
GGGTTTTGGACACTGGA- TTT                      --GTGGGCATCGGC in-frame
```

Mother Batch B

```
GGGTTTTGGACACTG---                          CCGTGGGCATCGGC in-frame
GGGTTTTGGACACTGGA-                          CCGTGGGCATCGGC x3 @1
GGGTTTTGGACACTGGA- CA                       CCGTGGGCATCGGC x2 @2
GGGTTTTGGACACTGGAA TACT                     ------GCATCGGC
GGGTTTTGGACACTGGAA (2)                      CCGTGGGCATCGGC x2 HDR in-frame
```

Mother Batch C

```
GGGTTTTGG---------                          -------CATCGGC
GGGTTTTGGA--------                          CCGTGGGCATCGGC
GGGTTTTGGACACTG--- (3)                      CCGTGGGCATCGGC HDR in-frame
GGGTTTTGGACACTGG-- GCAT                     -CGTGGGCATCGGC
GGGTTTTGGACACTGG-- (4)                      CCGTGGGCATCGGC in-frame
GGGTTTTGGACACTGGA-                          CCGTGGGCATCGGC x2 @1
GGGTTTTGGACACTGGA- TGC                      CCGTGGGCATCGGC
GGGTTTTGGACACTGGAA CATCGTGGGACCGTGGA        CCGTGGGCATCGGC
```

Mother Batch D

```
GGGTTTTGGACACTG---                          -------------C
GGGTTTTGGACACTGG--                          ------GCATCGGC x4
GGGTTTTGGACACTGG--                          -----GGCATCGGC
GGGTTTTGGACACTGGA-                          CCGTGGGCATCGGC x1 @1
```

Mother Batch E

```
GGGTTTTGGACACTGGA-                          CCGTGGGCATCGGC x3 @1
GGGTTTTGGAC------- GATG                     CCGTGGGCATCGGC in-frame
GGGTTTTGGACACTGGA- CA                       CCGTGGGCATCGGC @2
GGGTTTTGGACACTGGA- CCGTGGACCGTGGA           CCGTGGGCATCGGC
GGGTTTTGGACACTGGAA TGACGATG                 CCGTGGGCATCGGC
```

x# Indicates the number of males in which the allele sequence was found

@# Indicates a sequence that is identical to a sequence from a different mother

(1) Sequencing also included one fly with multiple alleles (r2m genetic mosaic)

(2) Large insertion (partial HDR of the drive allele 3' end up to the gRNA sequence cut site) =
CCGGTTTTAGAGCTAGAAATAGCAAGTTAAAATAAGGCTAGTCCGTTATCAACTTGAAAAAGTGGCACCGAGTCGGT
GCTTTTTTGCCTACCTGGAGCCTGAGAGTTGTTCAATAAAATAAAAATGTTTCGTTTTTTTGCTTTCGCCAGTATTT
ATTATTTTTCATCAATATGTATTCAATTTGGTATGTATTTAGTAATTGTAATATATAGACAATGGTTTTCCGTTGAC
GTACATACATCTGACGTGTGTTTATTTAGACATAATAGTTATGTTTTCACATCTTTTTAATGTTCGCTTAATGCGTA
TGCATCCTGCAGGACGCGT

(3) Large insertion (partial HDR of the drive allele 3' end) =
TGTGTGGACGTGTGTTTATTTAGACATAATAGTTATGTTTTCACATCTTTTTAATGTTCGCTTAATGCGTATGCATC
CTGCAGGACGCGT

(4) Large insertion = GTGGGCATCGTGGCATCGTGGCATGGACA

Resistance allele sequences from the *vasa* drive:

Red = gRNA Target Site

Orange = PAM

Blue = Insertion

\- = deletion

Wild type allele

```
TATTTGACCCTCA               GTGAATTGTGAATCATCG
```

Wild type male alleles

Mother Batch A

```
TATTTGA------               ----ATTGTGAATCATCG @1
TATTTGA------               -TGAATTGTGAATCATCG
TATTTGACCCTCA               -TGAATTGTGAATCATCG
TATTTGACCCTCA TCATTTGACCC   -----TTGTGAATCATCG
```

Mother Batch B

```
TATTTGA------               ----ATTGTGAATCATCG @1
TATTTGACCCT--               --GAATTGTGAATCATCG
TATTTGACCCTCA               ----ATTGTGAATCATCG
TATTTGACCCTCA T             -TGAATTGTGAATCATCG
```

Mother Batch C

```
TATTTGA------               ----ATTGTGAATCATCG @1
TATTTGACCCTCA               --GAATTGTGAATCATCG @2
TATTTGACCCT-- T             -TGAATTGTGAATCATCG @3
TATTTGACCCTCA TCATCGAATCATT --------TGAATCATCG
```

Mother Batch D

```
T------------               ------TGTGAATCATCG
TATTTGA------               -----TTGTGAATCATCG
TATTTGA------               ----ATTGTGAATCATCG @1
```

Mother Batch F

```
TATTTGACCCT--               -TGAATTGTGAATCATCG
TATTTGACCCT-- T             -TGAATTGTGAATCATCG @3
TATTTGACCCTCA TCAATCAATT    -TGAATTGTGAATCATCG
```

Mother Batch E

```
TATTTG-------               --------TGAATCATCG @4
TATTTGACCCT--               ------TGTGAATCATCG
TATTTGACCCTCA G             GTGAATTGTGAATCATCG
```

Mother Batch E

```
TATTTGA------               ----ATTGTGAATCATCG @1
TATTTGACCCTCA TT            -TGAATTGTGAATCATCG
```

Mother Batch G

```
TATTTGACCCTCA               --GAATTGTGAATCATCG @2
TATTTGACCCTCA T             ----ATTGTGAATCATCG
```

Mother Batch H

```
TATTTG-------               --------TGAATCATCG @4
TATTTGACCCTCA               ---AATTGTGAATCATCG @5
```

Others (each individual sequence from a male with a different mother)

```
TATTTGACCCTCA               ---AATTGTGAATCATCG x2 @5
TATTTGA------ CCCTCA        ---AATTGTGAATCATCG
TATTTGACCCTCA CCAAAT        ----ATTGTGAATCATCG
```

Yellow phenotype male alleles

Others (each individual sequence from a male with a different mother)

```
TATTTGACCCTCA               -----TTGTGAATCATCG
```

`x#` Indicates the number of males in which the allele sequence was found

`@#` Indicates a sequence that is identical to a sequence from a different mother
